# Comparison of four DNA extraction methods for 16s rRNA microbiota profiling of human faecal samples

**DOI:** 10.1186/s13104-023-06451-7

**Published:** 2023-08-11

**Authors:** James Sinclair, Nicholas P West, Amanda J Cox

**Affiliations:** 1 Menzies Health Institute Queensland, Griffith University, Parklands Drive, Southport, QLD 4215 Australia; 2 School of Pharmacy and Medical Sciences, Griffith University, Parklands Drive, Southport, QLD 4215 Australia

**Keywords:** Gut microbiota, Faecal samples, 16s rRNA, DNA extraction

## Abstract

**Objective:**

Growth in large population-based studies assessing contributions of the gut microbiota to health and disease requires high-throughput sample processing and analysis methods. This study assessed the impact that modifications to a commercially available magnetic bead based, semi-automated DNA extraction kit had on determination of microbial composition, relative to an established in-house method involving a combination of mechanical and chemical lysis. DNA was extracted from faecal samples from healthy adults (n = 12; 34–69 years), microbial composition was determined by V3-V4 16s rRNA sequencing and compared between extraction methods.

**Results:**

Diversity metrics did not differ between extraction methods. Differences in the relative abundance of key phyla, including a significantly lower abundance of the Firmicutes (p = 0.004) and higher relative abundance of the Bacteroidetes (p = 0.005) and Proteobacteria (p = 0.008) phyla were noted where the DNA extraction did not include additional chemical and mechanical lysis. Principal coordinate analysis of family and genera level data also suggested a potential for sample pre-processing to impact microbial composition. Observations of the potential for skewed microbial composition profiles from samples prepared using a semi-automated DNA extraction kit without additional sample pre-processing highlights a need for consideration of standardisation of methodological approaches to increase the comparability of microbial compositional data.

**Supplementary Information:**

The online version contains supplementary material available at 10.1186/s13104-023-06451-7.

## Introduction

There has been an increased number of studies exploring the contribution of the gut microbiota to health and disease over the past decade [1]. The potential for differences in the relative abundance of particular microbial species as well as the constitutive variety of bacterial species that contribute to human health outcomes continues to be of interest. In this context, recognition and standardisation of the numerous factors involved in accurately profiling the microbiome, needs to be addressed to support comparisons between studies, so that the potential utility of information from the volume of microbial composition data being generated is realised.

Factors that affect the downstream assessment of microbial composition, such as sample collection techniques, storage conditions, DNA extraction processing methods, selection of 16s rRNA hypervariable regions for library preparation and sequencing [2], and data analysis pipelines [3], have been previously established [4]. Variation in any of these methodological approaches have been shown to impact diversity metrics [5], detection of gram-positive or anaerobic bacteria [6, 7], and the estimation of low-abundant or rare taxa [8]. With an increase in large population-based studies the need for high-throughput sample collection, processing and analysis methods has led to growth in the range of commercially available sample collection and processing kits, some that use automated platforms to reduce processing times. The extent to which methodological variations may impact accurate determination of microbial composition requires further consideration.

The potential for different DNA extraction methods to contribute variation to assessment of microbial composition is documented in a range of sample types [9, 10] and strategies to improve both DNA yield and reduce the presence of inhibitory substances when extracting gut-derived sample material for microbial compositional profiling have been documented for almost two decades [11, 12]. Given the continued emergence of commercial DNA extraction kits and automated platforms, ongoing consideration of potential impacts of DNA extraction approaches on downstream microbial compositional profiles is needed. The aim of this study was to assess the effect that modifications to a commercially available magnetic bead based, semi-automated DNA extraction kit had on determination of microbial composition, relative to an established in-house method involving a combination of mechanical and chemical lysis.

## Main text

### Methods

This study involved a comparative analysis of four different DNA extraction methods for downstream assessment of faecal microbial composition. Samples (n = 12; 4 female, 8 male; age: 34–69 years) were collected using faecal collection kits which included a 70 mL faecal collection cup with scooped lid (Sarstedt, Australia) and flushable collection paper (Eiken Chemical, Tokyo, Japan) and were to be free of water and urine. Faecal samples were returned to the laboratory within 24 h of collection and stored at -80 °C until analysis. The study was undertaken in accordance with the Declaration of Helsinki. Individuals were otherwise healthy community-dwelling adults and provided written informed consent prior to participation. Ethical approval was provided by the Griffith University Human Research Ethics Committee (ref#: MED/19/15/HREC).

Upon thawing, faecal samples were homogenized in phosphate buffered saline, subsampled at equal volumes for extraction, and processed using one of the following DNA extraction methods: (i) an established in-house method involving repeated cycles of chemical and mechanical (bead beating) lysis, salt and alcohol precipitation and subsequent nucleic acid purification extraction as has been previously reported [11] (In-house); (ii) initial chemical and mechanical lysis steps consistent with the in-house protocol with processing using the Maxwell® RSC Faecal Microbiome DNA kit (Cat.# AS1700, Promega, Madison, USA) as per the manufacturers standard workflow (#TM640) (In-house + Maxwell); (iii) the Maxwell® RSC Faecal Microbiome DNA kit, following the manufacturers protocol for including an additional bead beating step (#PA663) preceding the standard workflow (#TM640) (Maxwell + bead-beating); (iv) Maxwell® RSC Faecal Microbiome DNA kit, following the standard workflow (#TM640) only (Maxwell). Chemical lysis used for methods (i) and (ii) above involved addition of a lysis buffer (500 mM NaCl, 50 mM Tris-HCl, pH 8.0, 50 mM ethylenediaminetetraacetic acid, and 4% sodium dodecyl sulfate) to homogenised faecal material and incubation at 70 °C consistent with the method of Yu et al. [11]. Mechanical lysis used for methods (i) and (ii) above involved addition of 0.1 and 1 mm zirconia beads (Daintree Scientific, Saint Helens, TAS, Australia) to homogenized faecal material mixed with lysis buffer, and homogenization using a Qiagen TissueLyser II (Qiagen, Hilden Germany).

DNA isolated using the different extraction methods was used for microbial compositional profiling with data generation and analysis as described previously [13]. Briefly this involved 16s rRNA gene sequencing of the V3-V4 region using universal primers (341 F: 5′-CCTACGGGNGGCWGCAG-3′; 805R: 5′-GAC TACHVGGGTATCTAATCC-3′), clustering of generated sequence reads into operational taxonomic units (OTU) at 97% identity level using the Quantitative Insights in Microbial Ecology (QIIME) Suite, and taxonomic identity assignment using a reference-based approach with the NCBI database of 16 S rRNA gene sequences. The relative abundance of prevalent taxa (present in greater than 75% of the samples) in the in-house method was compared pairwise to each other method using a Wilcoxon matched-pair signed rank test or the Kruskal-Wallis test for comparing multiple groups. Taxa considered not prevalent in the sample were not compared between methods. A principal coordinate analysis (PCoA) of Bray Curtis distances (relative abundance values) was used to explore the difference in the global microbial composition between the samples of each group; PERMANOVA analysis was completed using PERMANOVA + for PRIMER v7 (PRIMER-e Ltd, Plymouth, UK). Statistical significance was accepted at p < 0.05. For the microbial relative abundance comparisons Benjamini-Hochberg correct p-values are also reported to account for false discovery rate given the multiple comparisons performed.

## Results

An average of 19,784 ± 3,694 reads per sample were used for taxa assignment. The total OTU count (~ 155 OTU; p = 0.94) and Shannon Diversity Index (p = 0.79) did not differ between DNA extraction methods (Table 1; Additional File 1). At the phyla level, five phyla were prevalent (Table 1). There were significant differences between the relative abundances of Bacteroidetes (p = 0.005), Firmicutes (0.004), and Proteobacteria (0.008) phyla between the Maxwell method compared to In-house (Additional File 1). The Cyanobacteria and Fusobacteria were not prevalent among groups and persisted at low relative abundance (< 0.001%; data not shown).

**Fig. 1:**
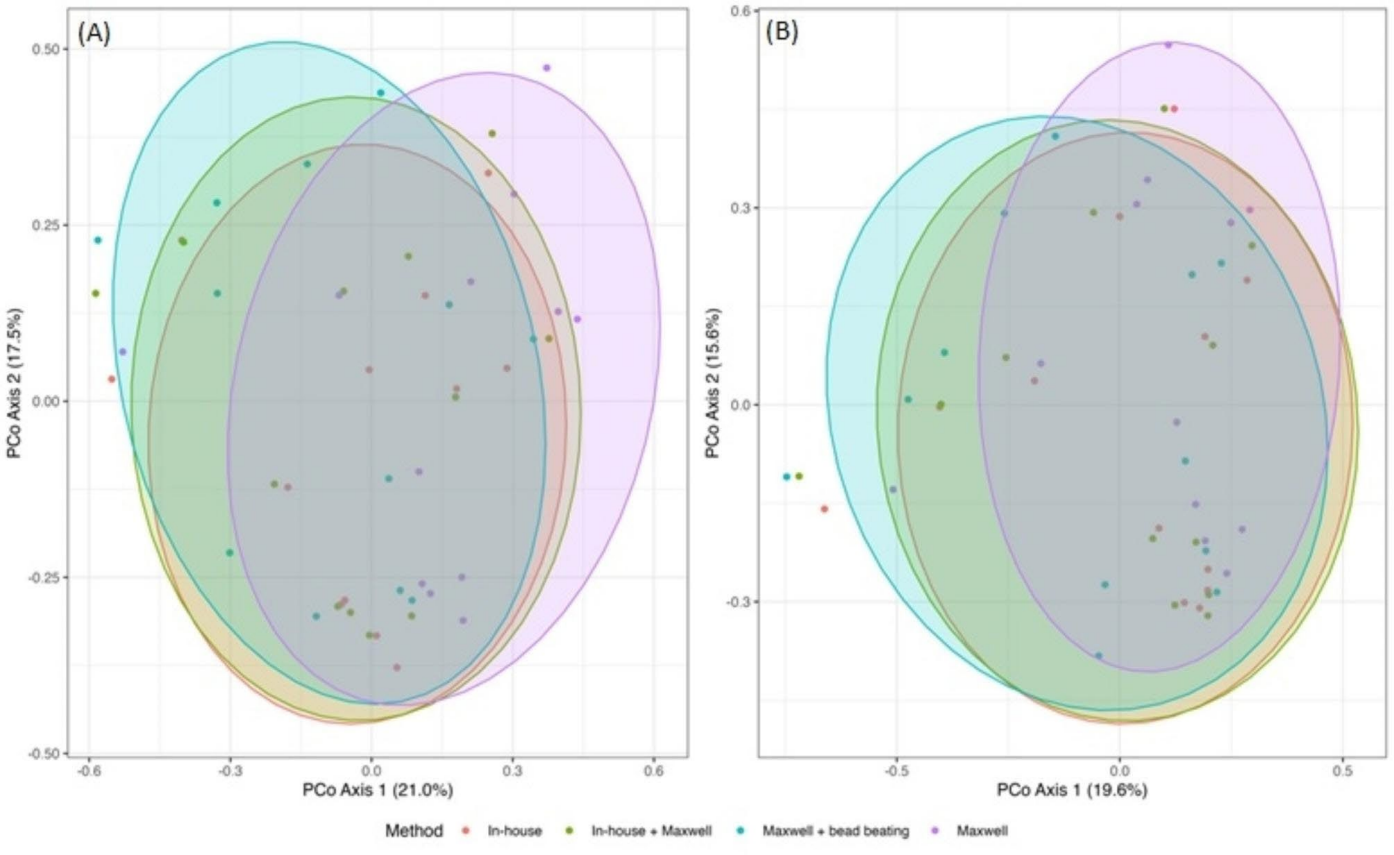
Principle coordinate analysis for (A) prevalent families (B) prevalent genera

At the Family level, 46 unique bacterial families were identified; 24 of these families were considered prevalent within the sample set (Additional File 2: Table S1). For three families (~ 13%) there were significant differences in relative abundance between Maxwell + bead beating and In-house methods, and there were 10 families (~ 42%) with significant differences in relative abundance between Maxwell and In-house (Additional File 2: Table S1). Using the prevalent bacterial families, a PCoA indicated that there was a trend for the microbial composition from the Maxwell method to differ from the In-house method (pseudo-F = 1.90, p = 0.09), but this trend was not evident when comparing the In-house method to the In-house + Maxwell (pseudo-F = 0.16, p = 0.98) and Maxwell + Bead beating (pseudo-F = 0.53, p = 0.80) methods (Fig. 1A).

At the Genus level, 146 unique bacterial genera were identified; 50 of these genera were considered prevalent within the sample set (Additional File 2: Table S2). For five genera (10%) there were significant differences in relative abundance between In-house + Maxwell and In-house methods; for nine genera (18%) there were significant differences in relative abundance between Maxwell + bead beating and In-house methods; and for 17 genera (34%) there were significant differences in relative abundance between Maxwell and the In-house methods. Using the prevalent genera, a PCoA indicated that there was a trend for the microbial composition from the Maxwell method to differ from the In-house method (pseudo-F = 1.71, p = 0.10), but this trend was not evident when comparing the In-house method to the In-house + Maxwell (pseudo-F = 0.10, p = 0.99) and Maxwell + bead-beating (pseudo-F = 0.66, p = 0.70) methods (Fig. 1B).

**Table 1:** Diversity metrics and relative abundance data for prevalent bacteria phyla for samples extracted using different DNA extraction methods. Data are presented as mean ± SD (median, range). P-values were determined using a Wilcoxon matched-pair signed rank test compared to In-house. Benjamini-Hochberg corrected p values are also presented [p=]

	In-house	In-house + Maxwell	Maxwell + bead beating	Maxwell
Alpha Diversity				
Richness	159.9 ± 44.16 (160, 84–233)	155.6 ± 44.4 (159, 85–240) p = 0.77	154.7 ± 45.7 (162, 78–226) p = 0.82	149.9 ± 41.2 (144, 71–225) p = 0.54
Shannon	4.76 ± 0.61 (4.82, 3.40–5.67)	4.73 ± 0.66 (4.79, 3.24–5.68) p = 0.93	4.77 ± 0.60 (4.80, 3.38–5.54) p = 1.0	4.53 ± 0.75 (4.61, 2.73–5.56) p = 0.38
**Phylum**				
Actinobacteria	3.4 ± 7.3 (0.59, 0.17–25.9)	2.7 ± 4.3 (0.63, 0.19–13.9) p = 0.88 [p = 0.93]	3.4 ± 4.7 (1.52, 0.45–16.7) p = 0.06 [p = 0.10]	2.3 ± 4.7 (0.42, 0.03–16.3) p = 0.01 [p = 0.15]
Bacteroidetes	28.7 ± 13.6 (31.6, 0.31–41.7)	29.6 ± 16.6 (28.2, 0.29–52.5) p = 0.94 [p = 0.97]	26.5 ± 19.8 (25.9, 0.08–52.4) p = 0.69 [p = 0.77]	42.6 ± 18.9 (47.7, 4.2–64.0) p = 0.005 [p = 0.01]
Firmicutes	55.4 ± 13.3 (58.6, 35.3–71.2)	55.1 ± 18.1 (56.6, 24.0-84.9) p = 1.0 [p = 1.0]	57.1 ± 19.4 (50.1, 28.1–85.4) p = 0.75 [p = 0.83]	36.8 ± 15.5 (33.4, 19.0-73.9) p = 0.004 [p = 0.01]
Proteobacteria	7.8 ± 10.4 (1.95, 0.39–32.1)	8.4 ± 12.3 (2.02, 0.49–41.3) p = 0.58 [p = 0.66]	9.3 ± 12.9 (2.22, 0.19–40.2) p = 0.14 [p = 0.19]	12.6 ± 15.4 (3.63, 0.5–51.3) p = 0.008 [p = 0.02]
Verrucomicrobia	4.7 ± 10.4 (0.07, 0.00-36.7)	4.1 ± 9.6 (0.08, 0-33.8) p = 0.21 [p = 0.27]	3.8 ± 9.5 (0.06, 0-33.4) p = 0.17 [p = 0.23]	5.6 ± 13.5 (0.08, 0-47.7) p = 0.37 [p = 0.44]

## Discussion

Despite the exponential growth in studies exploring the contribution of the gut microbiota to various states of health and disease, datasets are largely incompatible with each other, predominately due to differences in experimental protocols [14]. Standardization of methodological approaches to overcome this inherent variability between studies has been recognised by others [15] as a way to maximise the potential utility of the growing volume of composition data for use in meta-analyses to ascertain clear disease associations and treatment effects. Given the growing number of large population-based studies and need for high-throughput sample analysis, we explored the impacts that modifications to a commercially available DNA extraction kit had on determination of microbial composition.

Established in-house methods for faecal DNA extraction for microbial composition profiling involve a series of mechanical and chemical lysis steps [11] and extended workflows that are not always amenable to high-throughput sample analysis. Commercially available extraction kits for use on automated platforms offer the advantage of shorter processing times and higher throughput. However, the impact that the modified workflows may have on the completeness of compositional data cannot be discounted. Notably, bead-beating approaches have long been used to lyse the tough outer membrane of some gram-positive bacteria, that may otherwise be undisrupted by chemical lysis alone [16]. The outcome of repeated chemical and mechanical lysis steps in an established in-house method was compared to a single pass of chemical and/or mechanical lysis as part of a shorter semi-automated protocol. Global diversity metrics were not significantly different between methods, although a modest decrease in OTU count (~ 6%) was noted when using the commercial kit without additional sample pre-processing steps. Of particular note, the relative abundance of four key bacterial phyla were significantly different when using the commercial kit (without bead beating) relative to the in-house method, including a marked reduction (~ 33%) in the relative abundance of the Firmicutes phylum and a potential over-representation of the relative abundance of the Bacteroidetes phylum (~ 45% higher) as a possible consequence of compromised detection of gram-positive species skewing the broader community profile. That said, we did observe that some of the lower-order taxa within this phylum appeared to be more susceptible than others to compromised detection when using the Maxwell method, including the known butyrate producing Anaerobutyricum, Blautia, and Faecaliabactierum genera. The extent of these differences were ameliorated, in part, with the introduction of additional sample pre-processing steps prior to completion of the automated extraction protocol (“In-house + Maxwell”). These observations support the need for consideration of additional lysis steps if employing automated DNA extraction approaches for microbial compositional profiling.

## Limitations

This study is not without its limitations, employing a modest sample and comparing the existing in-house method to a single commercial kit only. We also reported data to the genus level only and so are unable to comment on how rare taxa may have been impacted or if greater read depth may have impacted outcomes for lower-level taxa. However, despite these limitations, outcomes clearly indicate that underrepresentation of some taxa is possible when using extraction methods that do not include mechanical lysis. This observation is crucial in the ongoing consideration of standardisation of methodological approaches to increase the comparability of microbial compositional profiling studies. DNA extraction methods employing a combination of both mechanical and chemical lysis (either in-house or via additional pre-processing steps prior to use of commercial kits) are considered preferable in achieving a more accurate microbial compositional profile.

### Electronic supplementary material

Below is the link to the electronic supplementary material.

Supplementary Material 1

Supplementary Material 2

## Data Availability

The datasets used and/or analysed during the current study are available from the corresponding author on reasonable request.

## Abbreviations

OTU: operational taxonomic units
QIIME: Quantitative Insights in Microbial Ecology
PCoA: principal coordinate analysis
