# Supplementary material for: Comparison of four DNA extraction methods for 16s rRNA microbiota profiling of human faecal samples
Source: BMC Res Notes. 2023 Aug 11;16:169. doi: 10.1186/s13104-023-06451-7 (PMC10422837; doi:10.1186/s13104-023-06451-7)

Additional File 1

(A) OTU, (B) Shannon diversity and (C) relative abundance prevalent phyla between DNA extraction methods.

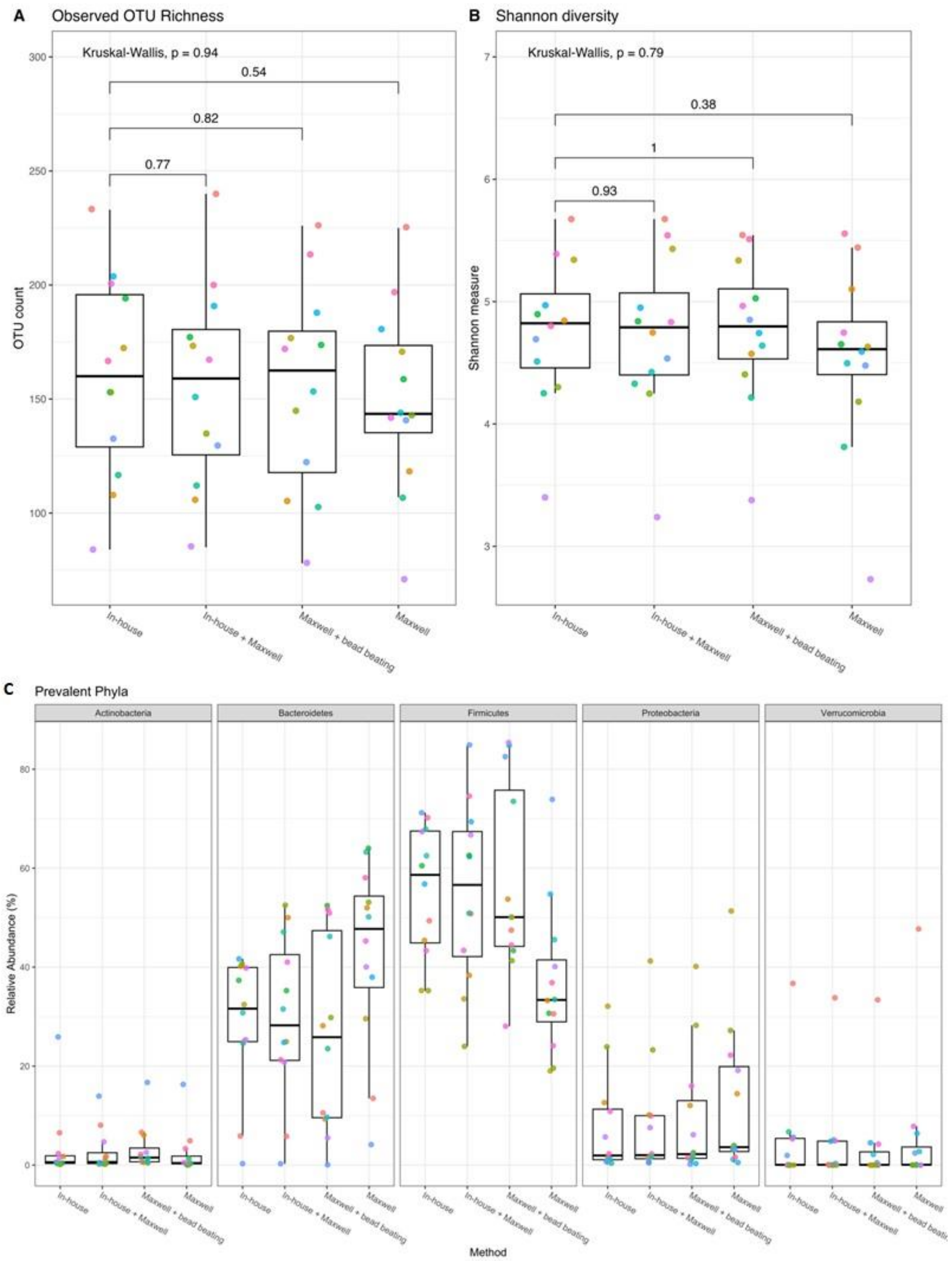

Supplement: Supplementary file 1 — Supplementary Material 1 [file 13104_2023_6451_MOESM1_ESM.pdf]
