# Supplementary material for: Comparison of four DNA extraction methods for 16s rRNA microbiota profiling of human faecal samples
Source: BMC Res Notes. 2023 Aug 11;16:169. doi: 10.1186/s13104-023-06451-7 (PMC10422837; doi:10.1186/s13104-023-06451-7)
Supplement: Supplementary file 2 — Supplementary Material 2 [file 13104_2023_6451_MOESM2_ESM.pdf]

Additional File 2

Table S1 - Relative abundance data for prevalent bacterial families for samples extracted using different DNA extraction methods. Data are presented as mean ± SD (median, range). P-values were determined using a Wilcoxon matched-pair signed rank test compared to In-house. Benjamini-Hochberg corrected p values are also presented [p=].

**Family**	**In-house**	**In-house + Maxwell**	**Maxwell + bead beating**	**Maxwell**
**Actinobacteria**				
Coriobacteriaceae	1.52 ± 4.78 (0.15, 0.00-16.7)	0.70 ± 1.97 (0.15, 0.00-6.94)<br>p=0.14 [p=0.22]	0.95 ± 2.15 (0.36, 0.00-7.71)<br>p=0.11 [p=0.19]	0.72 ± 1.99 (0.11, 0.00-7.01)<br>p=0.72 [p=0.78]
Eggerthellaceae	0.27 ± 0.42 (0.13, 0.02-1.51)	0.56 ± 1.32 (0.13, 0.04-4.70)<br>p=0.14 [p=0.21]	0.45 ± 0.70 (0.21, 0.05-2.58)<br>**p=0.03** [p=0.07]	0.31 ± 0.58 (0.09, 0.00-2.06)<br>p=0.69 [p=0.75]
**Bacteroidetes**				
Bacteroidaceae	8.65 ± 8.35 (5.15, 0.13-25.5)	8.72 ± 9.48 (5.41, 0.10-33.4)<br>p=0.75 [p=0.80]	6.60 ± 7.20 (3.69, 0.04-22.5)<br>p=0.31 [p=0.41]	10.87 ± 8.51 (10.0, 1.16-32.2)<br>**p=0.02 [p=0.05]**
Odoribacteraceae	0.31 ± 0.29 (0.27, 0.00-0.96)	0.31 ± 0.30 (0.30, 0.00-1.01)<br>p=0.72 [p=0.78]	0.30 ± 0.35 (0.15, 0.00-1.11)<br>p=0.80 [p=0.85]	0.53 ± 0.51 (0.45, 0.00-1.53)<br>**p=0.04** [p=0.08]
Rikenellaceae	5.01 ± 4.15 (4.16, 0.02-13.3)	4.83 ± 3.92 (4.00, 0.00-12.8)<br>p=0.35 [p=0.44]	2.88 ± 2.05 (2.67, 0.00-6.52)<br>p=0.18 [p=0.27]	7.25 ± 6.29 (5.27, 0.21-21.5)<br>**p=0.02 [p=0.05]**
Tannerellaceae	2.14 ± 2.31 (1.34, 0.00-7.07)	2.63 ± 3.29 (1.39, 0.00-11.1)<br>p=0.51 [p=0.60]	1.82 ± 1.85 (1.20, 0.00-4.85)<br>p=0.96 [p=0.97]	3.23 ± 3.42 (2.04, 0.00-10.7)<br>**p=0.05** [p=0.09]
Unclassified Bacteroidales	9.07 ± 5.63 (8.07, 0.16-18.8)	9.16 ± 5.99 (8.91, 0.19-21.2)<br>p=0.69 [p=0.75]	10.5 ± 10.6 (6.33, 0.03-35.8)<br>p=0.94 [p=0.94]	13.7 ± 10.1 (10.5, 2.79-39.4)<br>**p=0.003 [p=0.01]**
**Firmicutes**				
Acidaminococcaceae	1.06 ± 0.96 (0.93, 0.00-2.52)	1.00 ± 0.96 (0.75, 0.00-2.55)<br>p=0.42 p=0.52]	0.93 ± 1.07 (0.51, 0.00-2.91)<br>p=0.29 [p=0.39]	1.42 ± 1.80 (0.74, 0.00-5.75)<br>p=0.59 [p=0.67]
Christensenellaceae	0.37 ± 0.80 (0.06, 0.00-2.53)	0.39 ± 0.81 (0.05, 0.00-2.39)<br>p=0.39 [p=0.48]	0.35 ± 0.74 (0.04, 0.00-2.28)<br>p=0.33 [p=0.44]	0.42 ± 0.90 (0.07, 0.00-3.03)<br>p=0.65 [p=0.72]
Clostridiaceae	0.53 ± 0.69 (0.42, 0.01-2.61)	0.43 ± 0.38 (0.43, 0.00-1.46)<br>p=0.48 [p=0.57]	0.55 ± 0.63 (0.42, 0.00-2.41)<br>p=0.48 [p=0.57]	0.26 ± 0.19 (0.26, 0.00-0.59)<br>p=0.07 [p=0.13]

Clostridiales Family XIII. Incertae Sedis	0.16 ± 0.14 (0.11, 0.01-0.50)	0.19 ± 0.21 (0.13, 0.00-0.77)<br>p=0.53 [p=0.61]	0.16 ± 0.13 (0.14, 0.01-0.52)<br>p=0.58 [p=0.66]	0.18 ± 0.20 (0.13, 0.00-0.69)<br>p=0.58 [p=0.66]
Erysipelotrichaceae	1.86 ± 1.95 (0.99, 0.14-5.40)	2.06 ± 2.76 (1.08, 0.07-9.67)<br>p=0.58 [p=0.66]	3.46 ± 4.54 (2.60, 0.21-16.7)<br>**p=0.02 [p=0.04]**	1.50 ± 2.65 (0.44, 0.04-9.22)<br>p=0.10 [p=0.17]
Eubacteriaceae	1.95 ± 1.88 (1.68, 0.01-5.60)	1.73 ± 1.59 (1.37, 0.03-4.94)<br>p=0.07 [p=0.13]	1.95 ± 2.08 (1.10, 0.02-6.62)<br>p=0.94 [p=0.94]	1.74 ± 1.73 (1.19, 0.02-4.45)<br>p=0.81 [p=0.85]
Lachnospiraceae	16.7 ± 9.01 (13.6, 5.77-34.6)	19.7 ± 14.8 (13.4, 7.54-59.6)<br>p=0.31 [p=0.41]	22.7 ± 14.5 (18.6, 6.68-58.0)<br>p=0.08 [p=0.15]	11.1 ± 8.65 (7.58, 4.25-36.1)<br>**p=0.005 [p=0.02]**
Oscillospiraceae	3.47 ± 2.58 (3.09, 0.24-7.38)	2.91 ± 2.23 (2.14, 0.26-6.82)<br>p=0.58 [p=0.66]	2.77 ± 2.40 (2.49, 0.32-8.04)<br>p=0.27 [p=0.37]	4.03 ± 3.34 (2.86, 0.58-11.5)<br>p=0.31 [p=0.41]
Peptostreptococcaceae	0.21 ± 0.24 (0.11, 0.00-0.72)	0.30 ± 0.44 (0.15, 0.00-1.62)<br>p=0.88 [p=0.90]	0.35 ± 0.38 (0.26, 0.00-1.30)<br>p=0.07 [p=0.13]	0.16 ± 0.37 (0.06, 0.00-1.33)<br>p=0.16 [p=0.24]
Ruminococcaceae	19.4 ± 5.15 (18.2, 10.2-28.6)	16.0 ± 6.61 (15.2, 8.69-31.7)<br>p=0.16 [p=0.24]	14.3 ± 5.63 (14.4, 4.24-23.1)<br>**p=0.005 [p=0.02]**	8.11 ± 3.54 (7.56, 2.10-15.4)<br>**p=0.002 [p=0.01]**
Streptococcaceae	0.58 ± 1.14 (0.09, 0.00-4.04)	1.04 ± 2.81 (0.13, 0.00-9.90)<br>p=0.86 [p=0.89]	1.17 ± 2.64 (0.25, 0.01-9.36)<br>p=0.10 [p=0.17]	0.31 ± 0.57 (0.05, 0.00-1.97)<br>p=0.18 [p=0.27]
Unclassified Clostridiales	2.13 ± 1.80 (1.44, 0.04-6.47)	2.04 ± 1.92 (1.37, 0.04-7.12)<br>p=0.27 [p=0.37]	2.19 ± 2.04 (1.68, 0.02-7.17)<br>p=0.94 [p=0.94]	2.23 ± 2.32 (1.63, 0.04-8.03)<br>p=0.88 [p=0.90]
Veillonellaceae	4.27 ± 4.67 (2.22, 0.00-12.6)	3.81 ± 4.15 (1.70, 0.00-11.4)<br>p=0.15 [p=0.24]	3.59 ± 5.25 (1.04, 0.00-16.2)<br>p=0.42 [p=0.52]	2.82 ± 3.80 (1.43, 0.00-13.2)<br>p=0.42 [p=0.52]
**Proteobacteria**				
Desulfovibrionaceae	0.35 ± 0.49 (0.11, 0.00-1.32)	0.42 ± 0.58 (0.11, 0.00-1.68)<br>p=0.33 [p=0.43]	0.43 ± 0.65 (0.10, 0.00-1.76)<br>p=0.48 [p=0.57]	0.85 ± 1.14 (0.20, 0.00-3.05)<br>**p=0.03** [p=0.06]
Enterobacteriaceae	7.17 ± 10.6 (1.14, 0.00-32.1)	7.67 ± 12.4 (1.09, 0.00-41.3)<br>p=0.72 [p=0.78]	8.54 ± 13.21 (0.41, 0.00-40.2)<br>p=0.48 [p=0.57]	11.2 ± 16.0 (1.12, 0.00-51.3)<br>**p=0.05** [p=0.10]
Sutterellaceae	0.22 ± 0.29 (0.15, 0.00-0.97)	0.26 ± 0.44 (0.15, 0.00-1.58)<br>p=0.86 [p=0.89]	0.28 ± 0.31 (0.17, 0.00-0.80)<br>p=0.37 [p=0.48]	0.49 ± 0.58 (0.23, 0.00-1.58)<br>**p=0.01 [p=0.03]**
**Verrucomicrobia**				
Akkermansiaceae	4.71 ± 10.4 (0.07, 0.00-36.7)	4.09 ± 9.61 (0.08, 0.00-33.8)<br>p=0.21 [p=0.31]	3.75 ± 9.49 (0.06, 0.00-33.4)<br>p=0.17 [p=0.26]	5.61 ± 13.5 (0.08, 0.00-47.7)<br>p=0.37 [p=0.48]

Table S2 - Relative abundance data for prevalent bacterial genera for samples extracted using different DNA extraction methods. Data are presented as mean ± SD (median, range). P-values were determined using a Wilcoxon matched-pair signed rank test compared to In-house. Benjamini-Hochberg corrected p values are also presented [p=].

**Genus**	**In-house**	**In-house + Maxwell**	**Maxwell + bead beating**	**Maxwell**
**Actinobacteria**				
Collinsella	1.51 ± 4.78 (0.15, 0.00-16.7)	0.69 ± 1.97 (0.12, 0.00-6.94)<br>p=0.14 [p=0.22]	0.93 ± 2.15 (0.34, 0.00-7.71)<br>p=0.11 [p=0.19]	0.72 ± 1.99 (0.10, 0.00-7.01)<br>p=0.72 [p=0.78]
**Bacteroidetes**				
Alistipes	4.71 ± 4.34 (4.00, 0.02-13.3)	4.60 ± 4.11 (4.00, 0.00-12.8)<br>p=0.35 [p=0.44]	2.65 ± 2.06 (2.11, 0.00-6.52)<br>p=0.27 [p=0.37]	6.97 ± 6.48 (5.27, 0.21-21.5)<br>**p=0.02** [p=0.06]
Bacteroides	8.65 ± 8.35 (5.15, 0.05-25.5)	8.72 ± 9.48 (5.41, 0.05-33.4)<br>p=0.81 [p=0.85]	6.60 ± 7.21 (3.69, 0.02-22.5)<br>p=0.31 [p=0.41]	10.8 ± 8.57 (10.04, 0.54-32.2)<br>**p=0.02** [p=0.06]
Parabacteroides	2.14 ± 2.31 (1.34, 0.00-7.07)	2.63 ± 3.29 (1.39, 0.00-11.1)<br>p=0.51 [p=0.60]	1.82 ± 1.85 (1.20, 0.00-4.85)<br>p=0.96 [p=0.97]	3.23 ± 3.42 (2.04, 0.00-10.7)<br>**p=0.05** [p=0.10]
Phocaeicola	9.07 ± 5.63 (8.07, 0.16-18.8)	9.16 ± 5.99 (8.91, 0.19-21.2)<br>p=0.69 [p=0.76]	10.5 ± 10.6 (6.33, 0.03-35.8)<br>p=0.94 [p=0.95]	13.8 ± 10.1 (10.5, 2.79-39.4)<br>**p=0.003 [p=0.02]**
**Firmicutes**				
Agathobaculum	0.38 ± 0.58 (0.23, 0.01-2.13)	0.44 ± 0.67 (0.26, 0.01-2.49)<br>**p=0.04** [p=0.09]	0.37 ± 0.39 (0.31, 0.00-1.42)<br>p=0.94 [p=0.95]	0.14 ± 0.21 (0.09, 0.01-0.78)<br>**p=0.01 [p=0.02]**
Aminipila	0.06 ± 0.11 (0.02, 0.00-0.41)	0.09 ± 0.18 (0.03, 0.00-0.65)<br>p=0.37 [p=0.48]	0.08 ± 0.13 (0.04, 0.00-0.45)<br>p=0.13 [p=0.21]	0.05 ± 0.08 (0.02, 0.00-0.24)<br>p=1.00 [p=1.00]
Anaerobutyricum	0.46 ± 0.47 (0.36, 0.00-1.46)	1.06 ± 1.38 (0.29, 0.00-3.82)<br>p=0.11 [p=0.18]	1.43 ± 1.98 (0.41, 0.00-6.59)<br>**p=0.04** [p=0.09]	0.05 ± 0.10 (0.01, 0.00-0.34)<br>**p=0.003 [p=0.02]**
Anaeromassilibacillus	0.11 ± 0.15 (0.06, 0.01-0.55)	0.09 ± 0.09 (0.08, 0.00-0.30)<br>p=0.43 [p=0.53]	0.09 ± 0.11 (0.07, 0.00-0.35)<br>p=0.64 [p=0.72]	0.12 ± 0.15 (0.07, 0.01-0.51)<br>p=0.94 [p=0.95]
Anaerostipes	0.44 ± 0.79 (0.17, 0.00-2.83)	0.56 ± 0.93 (0.12, 0.01-3.20)<br>p=0.18 [p=0.27]	0.88 ± 1.35 (0.15, 0.01-4.55)<br>**p=0.03** [p=0.06]	0.17 ± 0.36 (0.02, 0.00-1.11)<br>p=0.18 [p=0.27]
Anaerotignum	0.25 ± 0.36 (0.14, 0.00-1.16)	0.24 ± 0.33 (0.16, 0.00-1.14)<br>p=0.24 [p=0.34]	0.12 ± 0.12 (0.10, 0.00-0.35)<br>p=0.11 [p=0.19]	0.25 ± 0.34 (0.12, 0.00-1.01)<br>p=0.72 [p=0.78]

Anaerotruncus	0.03 ± 0.03 (0.02, 0.00-0.13)	0.03 ± 0.04 (0.02, 0.00-0.15)<br>p=0.25 [p=0.34]	0.04 ± 0.05 (0.03, 0.00-0.20)<br>p=0.11 [p=0.18]	0.06 ± 0.08 (0.04, 0.00-0.29)<br>**p=0.01 [p=0.04]**
Blautia	4.10 ± 4.23 (2.02, 0.47-11.8)	5.76 ± 7.45 (1.78, 0.69-25.9)<br>**p=0.04** [p=0.09]	7.78 ± 7.74 (3.92, 0.71-24.9)<br>**p=0.01 [p=0.04]**	1.85 ± 2.28 (1.01, 0.07-6.42)<br>**p=0.005 [p=0.02]**
Christensenella	0.37 ± 0.80 (0.06, 0.00-2.53)	0.39 ± 0.81 (0.05, 0.00-2.39)<br>p=0.39 [p=0.49]	0.35 ± 0.74 (0.04, 0.00-2.28)<br>p=0.33 [p=0.43]	0.42 ± 0.90 (0.07, 0.00-3.03)<br>p=0.65 [p=0.72]
Coprococcus	2.14 ± 2.12 (1.07, 0.00-5.61)	2.38 ± 2.52 (1.00, 0.00-6.92)<br>p=0.33 [p=0.43]	2.63 ± 3.04 (1.64, 0.00-9.16)<br>p=0.72 [p=0.78]	0.99 ± 1.06 (0.56, 0.00-3.36)<br>**p=0.05** [p=0.10]
Dorea	1.40 ± 2.06 (0.60, 0.26-7.40)	1.88 ± 3.29 (0.93, 0.26-12.1)<br>p=0.35 [p=0.44]	2.24 ± 2.45 (1.71, 0.19-9.05)<br>**p=0.01 [p=0.04]**	0.87 ± 1.06 (0.31, 0.06-3.24)<br>**p=0.03** [p=0.07]
Emergencia	0.04 ± 0.04 (0.03, 0.00-0.12)	0.04 ± 0.04 (0.02, 0.00-0.11)<br>p=0.72 [p=0.78]	0.03 ± 0.03 (0.01, 0.00-0.11)<br>p=0.24 [p=0.34]	0.04 ± 0.05 (0.01, 0.00-0.12)<br>p=0.77 [p=0.82]
Enterocloster	0.15 ± 0.18 (0.07, 0.00-0.61)	0.14 ± 0.21 (0.06, 0.00-0.76)<br>p=0.66 [p=0.73]	0.08 ± 0.10 (0.04, 0.00-0.32)<br>**p=0.02 [p=0.05]**	0.12 ± 0.23 (0.03, 0.00-0.81)<br>p=0.35 [p=0.44]
Erysipelatoclostridium	0.82 ± 1.12 (0.36, 0.12-3.30)	0.71 ± 0.90 (0.46, 0.05-3.04)<br>p=0.43 [p=0.53]	1.11 ± 1.14 (0.70, 0.19-3.53)<br>**p=0.006 [p=0.02]**	0.31 ± 0.38 (0.20, 0.02-1.37)<br>**p=0.003 [p=0.02]**
Eubacterium	1.95 ± 1.87 (1.68, 0.01-5.60)	1.72 ± 1.58 (1.37, 0.03-4.90)<br>p=0.07 [p=0.13]	1.95 ± 2.08 (1.10, 0.02-6.62)<br>p=0.94 [p=0.95]	1.73 ± 1.73 (1.18, 0.02-4.45)<br>p=0.75 [p=0.81]
Faecalibacterium	11.1 ± 6.02 (11.4, 1.25-22.0)	9.70 ± 6.59 (9.23, 0.89-26.2)<br>p=0.35 [p=0.44]	5.40 ± 4.36 (3.77, 0.49-13.5)<br>**p=0.002 [p=0.02]**	2.00 ± 1.67 (1.75, 0.69-6.82)<br>**p=0.002 [p=0.02]**
Flavonifractor	0.24 ± 0.38 (0.03, 0.00-1.24)	0.17 ± 0.27 (0.02, 0.01-0.73)<br>p=0.07 [p=0.13]	0.18 ± 0.26 (0.03, 0.01-0.74)<br>p=0.69 [p=0.76]	0.24 ± 0.36 (0.05, 0.00-1.01)<br>p=0.58 [p=0.67]
Flintibacter	1.40 ± 1.11 (1.09, 0.01-4.03)	1.32 ± 1.13 (1.05, 0.01-4.12)<br>p=0.48 [p=0.58]	1.21 ± 1.00 (1.24, 0.01-3.85)<br>p=0.69 [p=0.76]	1.36 ± 1.27 (1.21, 0.02-4.72)<br>p=0.94 [p=0.95]
Fusicatenibacter	0.49 ± 0.54 (0.27, 0.00-1.39)	0.46 ± 0.46 (0.26, 0.00-1.17)<br>p=0.72 [p=0.78]	0.67 ± 0.86 (0.41, 0.00-2.99)<br>p=0.37 [p=0.48]	0.22 ± 0.23 (0.10, 0.00-0.58)<br>**p=0.04** [p=0.09]
Gemmiger	4.03 ± 6.06 (2.13, 0.00-19.9)	2.46 ± 2.95 (1.70, 0.00-10.0)<br>**p=0.01 [p=0.04]**	3.85 ± 4.41 (2.51, 0.00-14.9)<br>p=0.79 [p=0.84]	1.02 ± 0.72 (1.01, 0.00-2.37)<br>**p=0.01 [p=0.04]**
Hespellia	0.15 ± 0.18 (0.07, 0.03-0.60)	0.16 ± 0.22 (0.05, 0.01-0.62)<br>p=0.58 [p=0.67]	0.24 ± 0.28 (0.11, 0.02-0.82)<br>p=0.06 [p=0.11]	0.11 ± 0.17 (0.05, 0.01-0.59)<br>p=0.16 [p=0.24]
Ihubacter	0.06 ± 0.07 (0.02, 0.00-0.22)	0.05 ± 0.07 (0.02, 0.00-0.22)<br>p=0.66 [p=0.73]	0.04 ± 0.05 (0.03, 0.00-0.17)<br>p=0.42 [p=0.52]	0.08 ± 0.14 (0.02, 0.00-0.45)<br>p=1.00 [p=1.00]

Intestinimonas	0.38 ± 0.80 (0.04, 0.00-2.34)	0.40 ± 0.89 (0.04, 0.00-2.87)<br>p=0.72 [p=0.78]	0.55 ± 1.14 (0.05, 0.00-3.01)<br>p=0.65 [p=0.72]	0.62 ± 1.31 (0.05, 0.00-3.57)<br>p=0.39 [p=0.49]
Kineothrix	0.97 ± 0.53 (1.00, 0.35-2.12)	0.91 ± 0.59 (0.64, 0.26-2.13)<br>p=0.69 [p=0.76]	0.81 ± 0.55 (0.65, 0.30-2.04)<br>p=0.12 [p=0.19]	0.98 ± 1.07 (0.57, 0.09-3.84)<br>p=0.31 [p=0.41]
Lachnoclostridium	0.73 ± 0.75 (0.32, 0.06-1.96)	0.64 ± 0.74 (0.21, 0.04-2.10)<br>**p=0.05 [p=0.10]**	0.46 ± 0.62 (0.20, 0.03-2.08)<br>**p=0.03 [p=0.07]**	1.47 ± 3.39 (0.14, 0.01-11.6)<br>p=0.21 [p=0.30]
Lachnospira	0.50 ± 0.62 (0.31, 0.00-2.16)	0.47 ± 0.53 (0.30, 0.00-1.75)<br>p=0.79 [p=0.84]	0.40 ± 0.45 (0.30, 0.00-1.64)<br>p=0.08 [p=0.13]	0.53 ± 0.76 (0.26, 0.00-2.61)<br>p=0.79 [p=0.84]
Lacrimispora	0.28 ± 0.26 (0.17, 0.01-0.72)	0.31 ± 0.32 (0.16, 0.01-1.02)<br>p=0.88 [p=0.90]	0.25 ± 0.29 (0.12, 0.00-0.81)<br>p=0.21 [p=0.30]	0.34 ± 0.50 (0.14, 0.01-1.73)<br>p=0.94 [p=0.95]
Mediterraneibacter	1.53 ± 1.49 (0.82, 0.22-4.34)	1.68 ± 1.82 (1.01, 0.22-6.22)<br>p=0.94 [p=0.95]	1.99 ± 2.33 (1.07, 0.36-8.46)<br>p=0.39 [p=0.49]	1.84 ± 1.84 (1.17, 0.29-5.68)<br>p=0.16 [p=0.24]
Monoglobus	0.22 ± 0.23 (0.13, 0.02-0.71)	0.22 ± 0.23 (0.10, 0.02-0.76)<br>p=0.64 [p=0.72]	0.27 ± 0.28 (0.19, 0.01-0.99)<br>p=0.21 [p=0.30]	0.19 ± 0.17 (0.15, 0.02-0.51)<br>p=0.81 [p=0.85]
Negativibacillus	0.23 ± 0.28 (0.15, 0.00-0.95)	0.17 ± 0.19 (0.10, 0.00-0.55)<br>p=0.51 [p=0.60]	0.31 ± 0.37 (0.23, 0.00-1.31)<br>p=0.09 [p=0.16]	0.42 ± 0.46 (0.24, 0.00-1.27)<br>p=0.07 [p=0.13]
Neglecta	0.38 ± 0.39 (0.28, 0.03-1.45)	0.37 ± 0.47 (0.20, 0.02-1.64)<br>p=1.00 [p=1.00]	0.50 ± 0.51 (0.21, 0.01-1.41)<br>p=0.43 [p=0.43]	0.27 ± 0.56 (0.07, 0.02-2.03)<br>p=0.06 [p=0.11]
Oscillibacter	3.47 ± 2.58 (3.09, 0.24-7.38)	2.91 ± 2.23 (2.14, 0.26-6.82)<br>p=0.58 [p=0.67]	2.77 ± 2.40 (2.49, 0.32-8.04)<br>p=0.27 [p=0.37]	4.03 ± 3.34 (2.86, 0.58-11.50)<br>p=0.31 [p=0.41]
Paludicola	0.05 ± 0.11 (0.02, 0.00-0.41)	0.05 ± 0.09 (0.03, 0.00-0.33)<br>p=0.44 [p=0.54]	0.03 ± 0.05 (0.01, 0.00-0.19)<br>p=0.42 [p=0.52]	0.05 ± 0.10 (0.01, 0.00-0.35)<br>p=0.65 [p=0.72]
Pseudoflavonifractor	0.16 ± 0.10 (0.12, 0.04-0.33)	0.11 ± 0.09 (0.08, 0.02-0.25)<br>**p=0.03 [p=0.06]**	0.13 ± 0.14 (0.08, 0.01-0.50)<br>p=0.10 [p=0.17]	0.21 ± 0.22 (0.18, 0.04-0.84)<br>p=0.69 [p=0.76]
Romboutsia	0.14 ± 0.14 (0.11, 0.00-0.42)	0.19 ± 0.23 (0.13, 0.00-0.81)<br>p=0.88 [p=0.90]	0.26 ± 0.26 (0.18, 0.00-0.75)<br>p=0.10 [p=0.17]	0.11 ± 0.25 (0.03, 0.00-0.89)<br>p=0.16 [p=0.24]
Roseburia	0.36 ± 0.45 (0.13, 0.00-1.38)	0.34 ± 0.38 (0.16, 0.00-1.15)<br>p=0.88 [p=0.90]	0.27 ± 0.34 (0.15, 0.00-1.05)<br>p=0.24 [p=0.34]	0.20 ± 0.22 (0.11, 0.00-0.61)<br>p=0.21 [p=0.30]
Ruminococcus	0.82 ± 0.71 (0.60, 0.00-2.23)	0.81 ± 0.79 (0.50, 0.00-2.49)<br>p=0.72 [p=0.78]	1.70 ± 1.80 (1.31, 0.00-5.35)<br>**p=0.01 [p=0.03]**	1.14 ± 1.45 (0.62, 0.00-5.05)<br>p=0.44 [p=0.54]
Ruthenibacterium	0.61 ± 1.08 (0.24, 0.04-3.90)	0.44 ± 0.62 (0.23, 0.05-2.24)<br>p=0.39 [p=0.49]	0.39 ± 0.57 (0.16, 0.03-2.13)<br>p=0.14 [p=0.21]	0.64 ± 0.90 (0.18, 0.05-2.57)<br>p=0.94 [p=0.95]

Sporobacter	0.23 ± 0.27 (0.12, 0.00-0.72)	0.21 ± 0.26 (0.14, 0.00-0.86)<br>p=0.79 [p=0.84]	0.27 ± 0.36 (0.17, 0.00-1.23)<br>p=0.42 [p=0.52]	0.35 ± 0.42 (0.10, 0.00-1.23)<br>p=0.11 [p=0.18]
Streptococcus	0.45 ± 0.71 (0.09, 0.00-2.47)	0.57 ± 1.20 (0.13, 0.00-4.27)<br>p=0.86 [p=0.89]	0.91 ± 1.80 (0.25, 0.00-6.34)<br>p=0.18 [p=0.27]	0.30 ± 0.57 (0.05, 0.00-1.97)<br>p=0.18 [p=0.27]
Unclassified Lachnospiraceae	2.34 ± 3.41 (1.32, 0.00-12.2)	2.22 ± 3.18 (1.05, 0.01-11.4)<br>p=0.75 [p=0.81]	1.93 ± 2.19 (1.05, 0.00-7.85)<br>p=0.72 [p=0.78]	0.92 ± 1.42 (0.60, 0.00-5.30)<br>**p=0.01 [p=0.03]**
Unclassified Ruminococcaceae	0.53 ± 0.95 (0.18, 0.05-3.46)	0.48 ± 0.99 (0.15, 0.05-3.57)<br>p=0.53 [p=0.62]	0.64 ± 1.38 (0.16, 0.06-4.96)<br>p=0.48 [p=0.58]	0.76 ± 1.32 (0.18, 0.06-4.62)<br>p=0.12 [p=0.19]
**Proteobacteria**				
Desulfovibrio	0.35 ± 0.49 (0.11, 0.00-1.32)	0.42 ± 0.58 (0.11, 0.00-1.68)<br>p=0.33 [p=0.43]	0.43 ± 0.65 (0.10, 0.00-1.76)<br>p=0.48 [p=0.58]	0.85 ± 1.14 (0.20, 0.00-3.05)<br>**p=0.03** [p=0.06]
Escherichia	7.17 ± 10.6 (1.13, 0.00-32.1)	7.67 ± 12.4 (1.09, 0.00-41.3)<br>p=0.86 [p=0.89]	8.53 ± 13.2 (0.41, 0.00-40.1)<br>p=0.48 [p=0.58]	11.16 ± 16.0 (1.12, 0.00-51.3)<br>**p=0.05** [p=0.10]
**Verrucomicrobia**				
Akkermansia	4.71 ± 10.4 (0.07, 0.00-36.7)	4.09 ± 9.61 (0.08, 0.00-33.8)<br>p=0.21 [p=0.30]	3.75 ± 9.49 (0.06, 0.00-33.4)<br>p=0.17 [p=0.26]	5.61 ± 13.54 (0.08, 0.00-47.7)<br>p=0.37 [p=0.48]
